# Music of Different Tones Maintains Intestinal Immunity by Regulating the Intestinal Barrier and Intestinal Microbiota

**DOI:** 10.3390/ijms26062482

**Published:** 2025-03-11

**Authors:** Yong Zhang, Minghang Chang, Hongyu Wang, Qiang Xue, Yuanyuan Liu, Haidong Wei, Jun Bao, Jianhong Li

**Affiliations:** 1 College of Life Science, Northeast Agricultural University, Harbin 150030, China; zy1225735339@163.com (Y.Z.); 18846016331@163.com (H.W.); 18845898165@163.com (Q.X.); lyy3648@163.com (Y.L.); weihaidongneau@163.com (H.W.); 2 College of Animal Science and Technology, Northeast Agricultural University, Harbin 150030, China; hi_xiaoxiao@sina.com; 3 Key Laboratory of Chicken Genetics and Breeding, Ministry of Agriculture and Rural Affairs, Harbin 150030, China

**Keywords:** music, intestinal barrier, intestinal microbiota, intestinal immunity

## Abstract

Music as an environmental factor can maintain intestinal health in animals, but it is unclear whether this effect is influenced by the tones of the music. In this study, 100 Kunming white mice were randomly divided into control group (C group) with no music, and three music groups were exposed to Mozart K.448 in D, A and G tone (D group, A group and G group), respectively. To study the effects of different tones of Mozart K.448 on intestinal barrier and intestinal microbiota, mice were given musical stimulation from 1 to 63 days of age. The results showed that no apparent abnormalities were observed in the structure of ileum among groups. The mRNA expression levels of genes related to intestinal physical barrier (Claudin-1, Claudin-12, ZO-2, Mucin2, ZO-1 and Claudin-5) were significantly higher in music groups than those in C group (*p* < 0.05), and the mRNA expression levels of intestinal barrier genes in D group were the highest (*p* < 0.05). The levels of intestinal mucosal permeability (DAO and D-lactate) in D group were significantly lower than those in other groups (*p* < 0.05). Ileum HSP60 mRNA level in D group were significantly lower than that in other groups (*p* < 0.05). The mRNA expression level of IgA was significantly higher in music groups than C group (*p* < 0.05). Additionally, the mRNA and protein expression levels of IgG were significantly higher in D group than other groups (*p* < 0.05). Music stimulation increased the abundance of beneficial microbiota, such as *Lactobacillus* and *Sporosarcina* (*p* < 0.05). Mozart K.448 can strengthen intestinal barrier function to reduce intestinal permeability and improve intestinal immunity, while also having a positive significance in promoting the colonization of beneficial intestinal microbiota. In addition, the effect of tone D was more significant.

## 1. Introduction

As a method of environmental enrichment, music offers the advantage of being low-cost and easy to administer. There is a growing interest in using music therapy to enhance the health and welfare of animals. Research has demonstrated that zebrafish become calmer and reduce the secretion of pro-inflammatory cytokines interleukin 1 beta (IL-1β) and Interferon-gamma (IFN-γ) in response to repeated musical stimulation [1]. Bowman et al. observed that dogs exhibited reduced standing time and increased resting time when exposed to classical and pop music [2]. Furthermore, the study noted that dogs emitted longer growling periods when subjected to heavy metal rock music compared to classical music. Studies with mice also have demonstrated that exposure to classical music stimulation can enhance immune function and prevent oxidative stress [3]. Therefore, music holds significant potential for development as it can influence animal behavior, reduce stress and enhance immunity.

Intestinal immunity is a crucial component of overall organismal immunity, largely reliant on the barrier function of the intestine. It is widely acknowledged that the intestinal barrier comprises physical, chemical and immune components. The tight junction (TJ) serves as the cornerstone of the physical barrier within the intestine, comprising a complex interplay of Claudins, Occludins and Zonula Occludens-1 (ZO-1) [4,5]. 

Intestinal mucosal epithelial cells play a vital role in forming the chemical barrier by secreting mucus, digestive juices and bacteriostatic substances [6]. Previous research has indicated that chronic noise stimulation at 95 dB in rats can significantly disrupt the chemical barrier, leading to a twofold rise in permeability to intact biomolecules [7]. A diverse array of immune cells and molecules diffuse throughout the intestinal mucosal epithelium and lamina propria, forming the intestinal immune barrier. B-lymphocytes are the primary cells involved in intestinal humoral immunity, secreting substantial quantities of Immunoglobulin A (IgA) and Immunoglobulin G (IgG) to combat pathogens [8,9]. Li et al. investigated the effects of Mozart K.448 on immunity in growing pigs and discovered that music stimulation enhanced immunity by increasing IgG, IL-2 and IFN-γ levels [10].

The intestinal microbiota plays a crucial role in regulating intestinal permeability by forming a structural barrier on the surface of intestinal mucosa. This barrier serves to prevent the invasive effects of foreign antigens, such as viruses, microbiota and food particles, thereby contributing to the maintenance of immune system homeostasis [11]. Studies have demonstrated that mice in a noisy environment have an increased ratio of *Firmicutes* to *Bacteroidetes* in intestine, leading to glycolipid metabolic disorders [12]. Additionally, Li et al. found that chronic noise exposure can reduce the abundance and diversity of intestinal microbiota, thereby contributing to the development of systemic inflammation [13]. In summary, sound can influence the balance of intestinal microbiota, but there is limited research on whether tone, as a sound element, can exert an impact on intestinal microbiota.

Music has the potential to reduce stress and induce relaxation, but it remains unclear how tones affect the intestinal health and microbiota of animals. Therefore, in this experiment, a musical environment was designed with the tones of A, G and D of Mozart’s K.448 to investigate the effects of different musical tones on the intestinal health of Kunming White (KM) mice and the molecular mechanisms involved.

## 2. Results

### 2.1. Histopathological Analysis of Ileum Tissues

**Figure 1:**
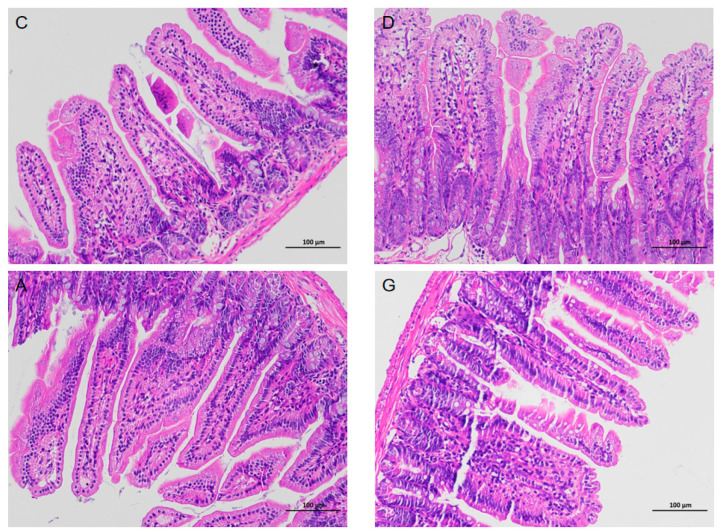
Histopathologic images of ileum tissues in each group. All images were magnified ×200. There were no obvious abnormalities in the structure of the ileum, and no obvious inflammatory changes were observed in each group. C, control group with no music stimulation; D, group exposed to Mozart K.448 in D tone; A, group exposed to Mozart K.448 in A tone; G, group exposed to Mozart K.448 in G tone.

The results of hematoxylin-eosin staining (HE) images of ileum tissue are depicted in Figure 1. The surface of the ileum in C, D, A and G groups exhibited a distribution of intestinal villi, with a single layer of columnar epithelium covering the surface. The epithelial cells displayed a striated edge, exhibiting normal morphology and structure. Cup-shaped cells were observed interspersed between the epithelial cells. At the base of the intestinal villi, the epithelium was invaginated to form the crypts of Lieberkühn. The mucosal muscular layer, composed of two layers of smooth muscle cells, separated the crypts of the intestinal villi from the submucosal layer. The submucosa was primarily composed of connective tissue, while the remainder of the intestinal wall consisted of the muscularis propria and layers of plasma membrane composed of smooth muscle cells. None of the aforementioned structures exhibited clear abnormalities, and no significant inflammatory changes were observed in any of the groups.

### 2.2. Immunofluorescence Staining of Ileum Physical Intestinal Barrier

**Figure 2:**
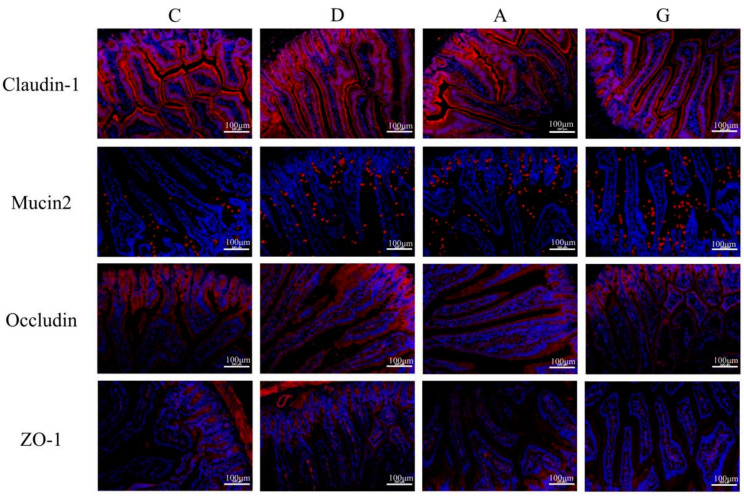
Immunofluorescence detection of Claudin-1, Mucin2, Occludin and ZO-1 expression in ileum tissue. Positive cells were stained red. The nucleus is stained blue. All images were magnified ×200. C, control group with no music stimulation; D, group exposed to Mozart K.448 in D tone; A, group exposed to Mozart K.448 in A tone; G, group exposed to Mozart K.448 in G tone; ZO-1, Zonula Occludens-1.

**Figure 3:**
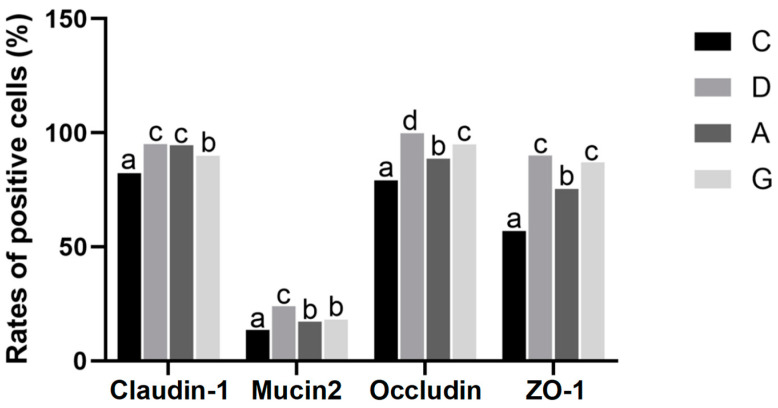
Positive cell rates of Claudin-1, Mucin2, Occludin and ZO-1. Different lower-case letters indicate a significant difference among different groups (*p* < 0.05). C, control group with no music stimulation; D, group exposed to Mozart K.448 in D tone; A, group exposed to Mozart K.448 in A tone; G, group exposed to Mozart K.448 in G tone. C, control group with no music stimulation; D, group exposed to Mozart K.448 in D tone; A, group exposed to Mozart K.448 in A tone; G, group exposed to Mozart K.448 in G tone; ZO-1, Zonula Occludens-1; ^a,b,c,d^ Bars with different superscripts in the same index are significantly different at *p* < 0.05.

The immunofluorescence staining of ileum physical intestinal barrier-associated proteins with positive cell rates is presented in Figure 2 and Figure 3. The positive cell rates of Claudin-1, Mucin2, Occludin and ZO-1 in the music groups were significantly higher than those in the C group (*p* < 0.05). Additionally, the positive cell rates of Claudin-1 in the D and A groups were significantly higher than those in the G group (*p* < 0.05), with no significant difference between the D and A groups (*p* > 0.05). Conversely, the positive cell rates of Mucin2 in the G and A groups were significantly lower than those in the D group (*p* < 0.05), with no significant difference between the G and A groups (*p* > 0.05). The rate of Occludin-positive cells in the D group was significantly higher than in the G and A group (*p* < 0.05), while the rate of Occludin-positive cells in the G group was significantly higher than in the A group (*p* < 0.05). Furthermore, the rate of ZO-1-positive cells was significantly higher in the D group and G group than in the C group and A group (*p* < 0.05), with no significant difference between the D group and G group (*p* > 0.05).

### 2.3. mRNA and Protein Expression Levels of Ileal Intestinal Barrier-Related Genes

**Figure 4:**
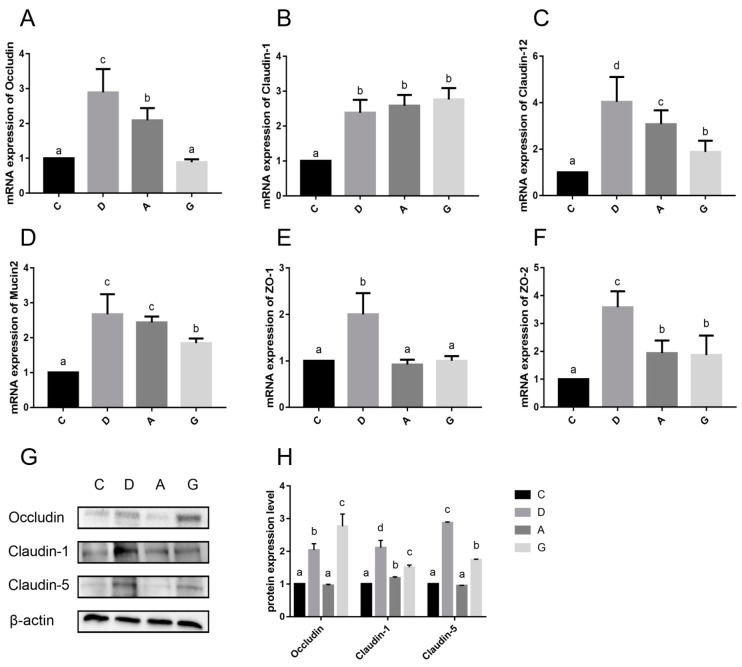
(**A**–**F**) Effects of different tones of music on the mRNA expression levels of ileum physical intestinal barrier genes in ileum tissue. (**G**,**H**) Effects of different tones of music on the protein expression levels of ileum physical intestinal barrier genes in ileum tissues. Data are shown as the mean ± SD. Different lower-case letters indicate a significant difference among different groups (*p* < 0.05). C, control group with no music stimulation; D, group exposed to Mozart K.448 in D tone; A, group exposed to Mozart K.448 in A tone; G, group exposed to Mozart K.448 in G tone; ZO-1, Zonula Occludens-1; ZO-2, Zonula Occludens-2; ^a,b,c,d^ Bars with different superscripts in the same index are significantly different at *p* < 0.05.

The mRNA expressions of ileum physical intestinal barrier genes are shown in Figure 4A–F. The mRNA expression levels of Claudin-1, Claudin-12, ZO-2 and Mucin2 in the music groups were significantly higher than those in the C group (*p* < 0.05). Additionally, the mRNA expression level of Occludin, Claudin-12, ZO-1 and ZO-2 in the D group was significantly higher than those in the other three groups (*p* < 0.05). Furthermore, the mRNA expression level of Occludin in the A group was significantly higher than that in the G group and C group (*p* < 0.05), whereas the mRNA expression level of Occludin in the G group did not significantly differ from that in the C group (*p* > 0.05). The mRNA expression level of Claudin-1 was not significantly different among the D, A and G groups (*p* > 0.05). Specifically, the mRNA expression level of Claudin-12 in the A group was significantly higher than that of the G group (*p* < 0.05), while the mRNA expression level of ZO-2 in the A group was not significantly different from that of the G group (*p* > 0.05). Furthermore, the mRNA expression levels of Mucin2 in the D group and A group were not significantly different (*p* > 0.05), but both were significantly higher than that in the G group (*p* < 0.05). Finally, the mRNA expression level of ZO-1 in the D group was significantly higher than that in the other three groups (*p* < 0.05), while no significant difference was observed among the C, A and G groups (*p* > 0.05).

The protein expressions of ileum physical intestinal barrier genes are shown in Figure 4G,H. The protein expression levels of Occludin, Claudin-1 and Claudin-5 were significantly higher in the D group and G group than in the C group and A group (*p* < 0.05). Additionally, the protein expression level of Occludin in the G group was significantly higher than that in the D group, whereas the protein expression levels of Claudin-1 and Claudin-5 were significantly lower than those in the D group (*p* < 0.05). Moreover, the protein expression level of Claudin-1 in the A group was significantly higher than that in the C group (*p* < 0.05), while the protein expression levels of Occludin and Claudin-5 were not significantly different between the C group and A group (*p* > 0.05).

### 2.4. The Level of Indicators Related to Ileal Barrier Permeability

**Figure 5:**
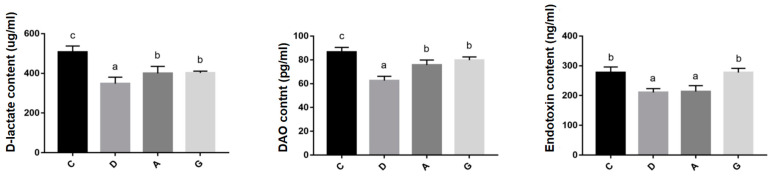
Effects of different tones of music on levels of indicators related to ileal barrier permeability. Data are shown as the mean ± SD. Different lower-case letters indicate a significant difference among different groups (*p* < 0.05). C, control group with no music stimulation; D, group exposed to Mozart K.448 in D tone; A, group exposed to Mozart K.448 in A tone; G, group exposed to Mozart K.448 in G tone; DAO, Diamine oxidase; ^a,b,c^ Bars with different superscripts in the same index are significantly different at *p* < 0.05.

The levels of ileal barrier permeability-related indicators are shown in Figure 5. The expression levels of D-lactate and DAO in music groups were significantly lower than those in C group (*p* < 0.05). Furthermore, the levels of D-lactate and DAO in D group were significantly lower than those in the A group and G group (*p* < 0.05), while the differences between the A group and G group were not significant (*p* > 0.05). Additionally, the expression levels of D-lactate and DAO in the D group were significantly lower than those in the A group and G group (*p* < 0.05), and the difference between the A group and G group was not significant (*p* > 0.05). Moreover, the expression levels of endotoxin in the D group and A group were significantly lower than those in the C group and G group (*p* < 0.05), while the difference between the D group and A group was not significant (*p* > 0.05), and the difference between the C group and G group was not significant (*p* > 0.05).

### 2.5. mRNA Expression Levels of Heat Shock Proteins

**Figure 6:**
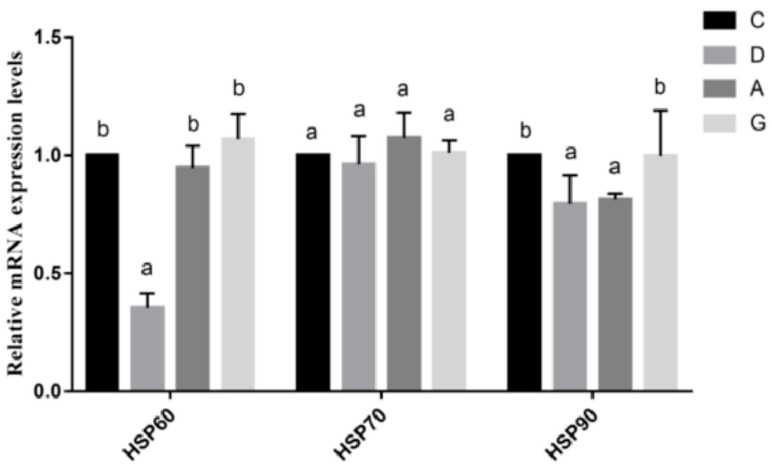
The effect of different tones of music on the mRNA expression levels of Heat Shock Protein-related genes in ileal tissues. Data are shown as the mean ± SD. Different lower-case letters indicate a significant difference among different groups (*p* < 0.05). C, control group with no music stimulation; D, group exposed to Mozart K.448 in D tone; A, group exposed to Mozart K.448 in A tone; G, group exposed to Mozart K.448 in G tone; HSP60, Heat Shock Protein 60; HSP70, Heat Shock Protein 70; HSP90, Heat Shock Protein 90; ^a,b^ Bars with different superscripts in the same index are significantly different at *p* < 0.05.

The mRNA expression levels of ileal Heat Shock Proteins (HSPs) are shown in Figure 6. The mRNA expression level of HSP60 in the D group was significantly lower than those in other groups (*p* < 0.05), while the mRNA expression levels of HSP60 were not significantly different among the C, A and G groups (*p* > 0.05). Additionally, there was no significant difference in the mRNA expression level of HSP70 in the music groups compared with the C group (*p* > 0.05). Moreover, the mRNA expression levels of HSP90 were significantly lower in the D group and A group than those in the C group and G group (*p* < 0.05). There was no significant difference in the mRNA expression level of HSP90 between the D group and A group, as well as between the C group and G group (*p* > 0.05).

### 2.6. mRNA and Protein Expression Levels of Ileal Immunoglobulin

**Figure 7:**
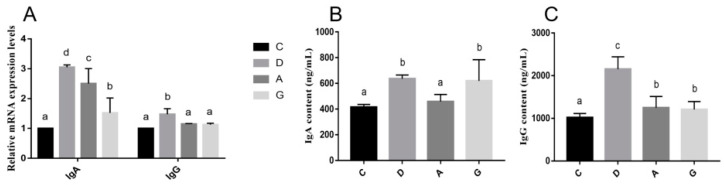
(**A**) Effects of different tones of music on mRNA expression levels of immunoglobulin-related genes in ileum. (**B**,**C**) Effects of different tones of music on the protein expression levels of immunoglobulin in ileum. Data are shown as the mean ± SD. Different lower-case letters indicate a significant difference among different groups (*p* < 0.05). C, control group with no music stimulation; D, group exposed to Mozart K.448 in D tone; A, group exposed to Mozart K.448 in A tone; G, group exposed to Mozart K.448 in G tone; IgA, Immunoglobulin A; IgG, Immunoglobulin G; ^a,b,c,d^ Bars with different superscripts in the same index are significantly different at *p* < 0.05.

The mRNA expression levels of ileal immunoglobulin are shown in Figure 7A. The mRNA expression level of IgA in the music groups was significantly higher than that in the C group (*p* < 0.05). Specifically, the mRNA expression level of IgA in the D group was significantly higher than that in the A group and G group (*p* < 0.05). Additionally, the mRNA expression level of IgG in the D group was significantly higher than that in the other three groups (*p* < 0.05), while the mRNA expression levels of IgG were not significantly different among the C, A and G groups (*p* > 0.05).

The protein expression levels of ileal immunoglobulin are shown in Figure 7B,C. The protein expression level of IgA was significantly higher in the D group and G group than in the C group and A group (*p* < 0.05). Specifically, there was no significant difference in the IgA protein expression level between the D group and G group (*p* > 0.05), and there was no significant difference between the C group and A group (*p* > 0.05). Additionally, the protein expression level of IgG in the D group was significantly higher than that in the other three groups (*p* < 0.05). Moreover, the protein expression level of IgG was significantly higher in the A group and G group than in the C group (*p* < 0.05), but there was no significant difference between the A group and G group (*p* > 0.05).

### 2.7. Analysis of Intestinal Microbiota

In this experiment, 16S rDNA sequencing was conducted on 24 samples, resulting in a total of 1,962,431 original sequences. After removing invalid sequences, 1,631,449 high-quality valid sequences were obtained, with an average of 67,977 valid sequences per sample. The average percentage of valid sequences across all samples was 83.25%. Furthermore, the average Q20 value was 96.25%, the average Q30 value was 90.75%, and the GC content percentage was 52.25%. These results indicate a high level of confidence in the sequencing outcomes.

**Figure 8:**
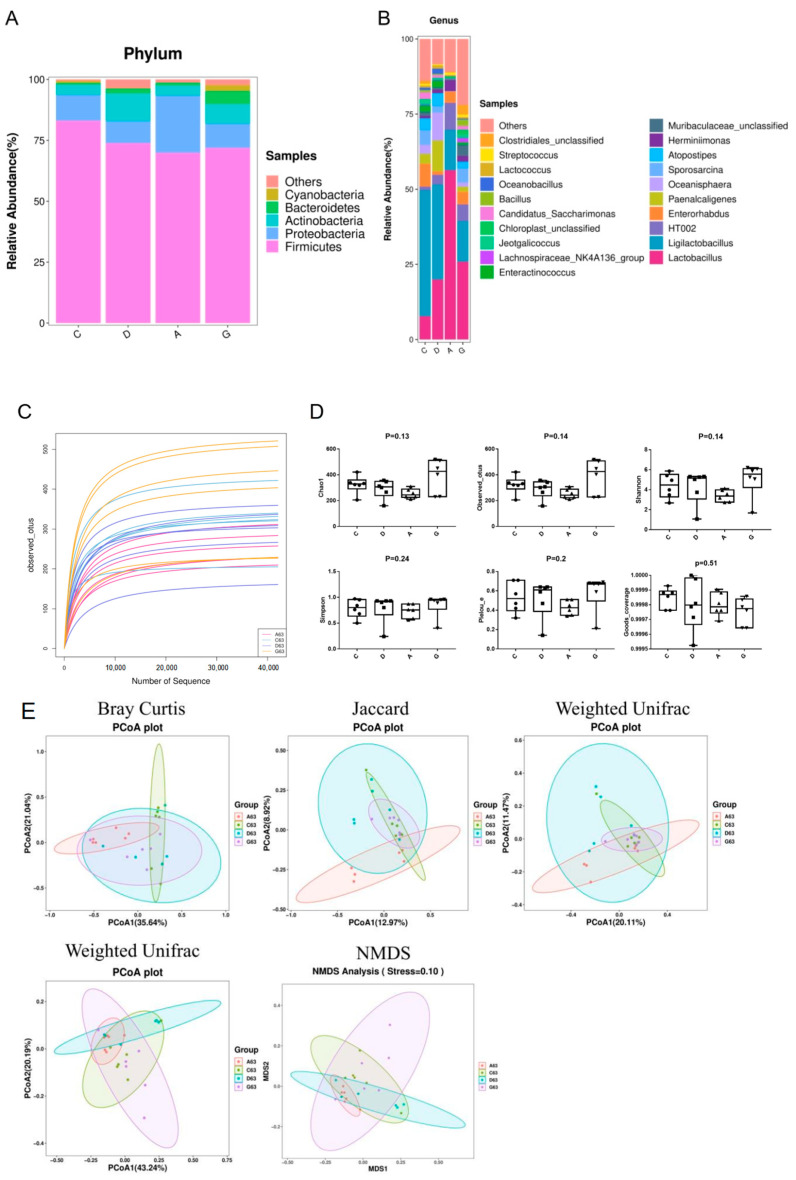
(**A**) Changes in the distribution of ileal microbiota at the phylum level. (**B**) Changes in the distribution of ileal microbiota at the genus level. (**C**) Dilution curve. (**D**) α-diversity analysis of ileal microbiota. The symbols in subfigure D represent individual data points for each group. (**E**) β-diversity analysis of ileal microbiota. C, control group with no music stimulation; D, group exposed to Mozart K.448 in D tone; A, group exposed to Mozart K.448 in A tone; G, group exposed to Mozart K.448 in G tone.

The relative abundance at the phylum levels is shown in Figure 8A. The distribution of microbiota at the phylum level in the C group was *Firmicutes* (83.06%), *Proteobacteria* (10.58%), *Actinobacteria* (4.22%) and *Bacteroidota* (0.69%). The distribution of microbiota at the phylum level in the D group was *Firmicutes* (73.83%), *Proteobacteria* (9.07%), *Actinobcteria* (11.36%) and *Bacteroidota* (2.04%). The distribution of microbiota at the phylum level in the A group was *Firmicutes* (69.87%), *Proteobacteria* (23.52%), *Actinobcteria* (4.13%) and *Bacteroidota* (1.16%). The distribution of microbiota at the phylum level in the G group was *Firmicutes* (71.86%), *Proteobacteria* (9.95%), *Actinobcteria* (8.08%) and *Bacteroidota* (5.35%). It is noteworthy that the abundance of *Proteobacteria* and *Actinobacteria* showed no significant differences among the four groups (*p* > 0.05). However, the abundance of *Bacteroidetes* was significantly higher in the G group compared to the other groups (*p* < 0.05), and the abundance of *Firmicutes* was significantly higher in the C group than in the other groups (*p* < 0.05).

The relative abundance at the genus levels is shown in Figure 8B. The distribution of microbiota at the genus level in the C group was *Ligilactobacillus* (41.95%), *Lactobacillus* (7.75%), *Enterorhabdus* (7.56%), *Paenalcaligenes* (3.37%), *Oceanisphaera* (2.91%), *Sporosarcina* (5.03%), *Atopostipes* (3.83%) and *Herminiimonas* (0.82%). The distribution of microbiota at the genus level in the D group was *Lactobacillus* (19.97%), *Ligilactobacillus* (31.62%), *Enterorhabdus* (1.14%), *Paenalcaligenes* (10.31%), *Oceanisphaera* (9.22%), *Sporosarcina* (2.27%), *Atopostipes* (4.23%) and *Herminiimonas* (1.19%). The distribution of microbiota at the genus level in the A group was *Lactobacillus* (56.34%), *Ligilactobacillus* (13.41%), *Enterorhabdus* (3.8%), *Paenalcaligenes* (0.02%), *Oceanisphaera* (0.02%), *Sporosarcina* (0.05%), *Atopostipes* (0.03%) and *Herminiimonas* (3.58%). The distribution of microbiota at the genus level in the G group was *Lactobacillus* (25.95%), *Ligilactobacillus* (13.52%), *Enterorhabdus* (4.14%), *Paenalcaligenes* (1.72%), *Oceanisphaera* (0.33%), *Sporosarcina* (4.85%), *Atopostipes* (2.13%) and *Herminiimonas* (1.9%). Statistical analysis indicates that the abundance of *Lactobacillus* in the A group was significantly higher than that in other groups (*p* < 0.05). The abundances of *Ligilactobacillus* in the A group and G group were significantly lower than those in the C group and D group (*p* < 0.05). The abundance of *Enterorhabdus* in the D group was significantly higher than that in the other groups (*p* < 0.05). It is worth noting that the abundances of *Paenalcaligenes*, *Sporosarcina* and *Atopostipes* in the A group were significantly lower than those in the other groups (*p* < 0.05).

The effect of different tones of music on the α-diversity of the ileum microbiota is shown in Figure 8C,D. Alpha diversity parameters include Chao1, Observed_otus, Goods coverage, Simpson, Pielou_e and Shannon. The observed sparsity curves of the bacterial species show a flat trend, indicating that the sequence depth was sufficient to cover almost all of the sequences. Chao1, Observed_otus, Goods coverage, Simpson, Pielou_e and Shannon were not significantly different among the four groups (*p* > 0.05).

The effect of different tones of music on the β-diversity of the ileum microbiota is shown in Figure 8E. In the results of PCoA, different colored dots represent different groups, and the proximity of the dots indicates a more similar composition of microbial structures between samples. In this study, the PCoA results based on Bray Curtis, Jaccard, Weighted Unifrac and Unweighted Unifrac showed that the samples were very close to each other among groups. The results indicated that microbial compositions were similar and that different tones of music did not significantly affect the structure of the intestinal microbiota of the mice (*p* > 0.05). NMDS is another important indicator of β-diversity, and the stress coefficient is used to evaluate the results of the analysis. In this study, the stress coefficient was 0.10, indicating that the difference in the species composition of the intestinal microbiota among the four groups was not significant (*p* > 0.05).

## 3. Discussion

The gut health of animals can be responded to by a combination of the intestinal barrier composed of tight junction proteins, the expression of intestinal immune cytokines and the gut microbiota. The integrity of the intestinal morphology and structure is the basis for its function. It has been shown that rats subjected to 15 min of 90 dB noise stimulation per day for 3 weeks exhibited abnormal detachment of intestinal mucosal epithelial cells and even detachment from the basement membrane, alongside significant oedema [14]. Conversely, an enriched environment has been shown to enhance rat intestinal barrier function by safeguarding the integrity of intestinal structure and modulating cytokine expression levels in intestinal tissues [15]. In our experiment, HE staining of ileal tissues revealed abundant and well-distributed ileal villi in mice exposed to music, with no evident pathological features observed. Therefore, we posit that music, as an environmental enrichment factor, does not detrimentally affect the morphological structure of the mice intestine. However, whether music will positively affect mice needs to be analyzed from a deeper perspective.

The intestinal barrier serves as a protective shield, preventing water and electrolyte loss while also thwarting the invasion of microorganisms and toxins from the lumen into the bloodstream. The TJ consists of the transmembrane proteins, occludins and claudins, along with periplasmic proteins such as ZO-1. These structures act as barriers by bridging cell gaps, thereby preventing harmful substances, such as pathogenic bacteria and toxins, from crossing the intestine into the body [16]. The intestinal mucus layer formed by Mucin2 protein shows dynamic interactions with the intestinal epithelial cells, microbiota and host immune defenses to maintain intestinal mucosal homeostasis. The diminishment of the TJ function and the reduced expression of Mucin2 protein both lead to impaired intestinal barrier function and increased intestinal mucosal permeability, which are considered to contribute to inflammatory bowel disease, obesity and metabolic disorders [17]. Studies have demonstrated that the enriched environment has a positive effect on the intestinal barrier function of juvenile Atlantic salmon, promoting their nutrient absorption and disease resistance [18]. Li’s research also revealed that exposure to low-decibel (65–75 dB) sound stimuli in a farm environment resulted in elevated levels of intestinal barrier protein expression in chicks. This suggests that mild auditory stimulation does not induce stress in the ileum of chicks and may potentially facilitate the development of the intestinal barrier [19]. The results of this study demonstrate that the mRNA expression levels of Claudin-1, Claudin-12, ZO-2 and Mucin2 in the music group were significantly higher than those in the C group. Additionally, the expression level of Claudin-1 protein in the music group was significantly higher than that in the C group. Immunofluorescence results also revealed that the positive cell rates of Claudin-1, Mucin2, Occludin and ZO-1 in the music group were markedly higher than those in the C group. These findings suggest that appropriate musical stimulation can maintain the integrity of the intestinal barrier in mice and prevent potential inflammatory damage to the intestinal mucosal tissue. Within the music group, we observed that the mRNA expression levels of Claudin-1, Claudin-12, ZO-2 and Mucin2 in the D group were significantly higher than those in the A and G groups. Additionally, the protein expression levels of Occludin and Claudin-5, as well as the positive cell rates of Occludin and ZO-1, were notably higher in the D and G groups compared to the A group. The results among the music groups indicate that specific musical tones, such as D or G, may selectively enhance the expression of genes associated with intestinal TJ, thereby improving intestinal barrier function. However, we observed that the mRNA expression and protein levels of Occludin in the A and G groups were not synchronized. This discrepancy may arise from post-transcriptional regulation or variations in translational efficiency, suggesting that the stability of Occludin protein levels could be modulated by post-translational modifications or degradation mechanisms [20]. In this study, we prioritized protein-level data to interpret the functionality of Occludin, as the integrity of tight junctions ultimately depends on the presence and localization of the protein rather than its mRNA [21].

DAO is a highly active enzyme found in mammalian intestinal villous cells. D-lactate is a byproduct of bacterial fermentation in the human gastrointestinal tract. Disruption of the intestinal barrier can lead to intestinal mucosal cell death and increased levels of DAO and D-lactic acid in the bloodstream [22]. Endotoxin, a toxic substance released by lysis of Gram-negative bacteria, serve as a potent stimulant of the immune response. When the intestinal barrier is compromised, endotoxin originating from intestinal microorganisms can enter the circulatory system, triggering an immune response [23]. Consequently, DAO, D-lactate and endotoxin are commonly used as indicators of intestinal permeability. Studies have shown that chronic noise exposure can increase intestinal permeability and induce multi-organ oxidative damage in mice, associated with intestinal stress syndrome [24]. In contrast, Orock et al. discovered enhanced intestinal permeability in the colons of rats raised in enriched environments [25]. The results of the present study indicated that the levels of D-lactate, DAO and endotoxin in serum were significantly higher in the C group than those in music groups.

Additionally, D-lactate and DAO levels were reduced in serum and were significantly lower in the D group than in the A group and G group. These findings suggested that music stimulation reduces intestinal permeability, aligning with previous research linked to enriched environments with improved gut permeability in mice [26]. Furthermore, this study found that the expression of intestinal barrier-related genes (such as Claudin-1, ZO-1, Occludin and Mucin2) in the D group was significantly higher than that in the A and G groups, while the levels of intestinal permeability indicators (DAO and D-lactate) were also lower in the D group. These results suggest that Mozart’s K.448 in D tone has a more significant effect on enhancing intestinal barrier function and reducing intestinal permeability. This phenomenon may be related to the frequency characteristics of D major music and its unique impact on the physiological state of mice.

HSPs are integral components of the cellular molecular chaperone system, present in all cells with a high degree of conservation between species. Outer cell membrane-bound HSPs mediate the immune function of cells, thereby triggering immune responses within the immune system, enabling cells to survive under stressful conditions [27]. Several studies have confirmed that broilers raised in enriched environments or fed selenium-enriched diets exhibit reduced levels of HSPs and diminished stress injury [28]. To investigate whether music can alleviate intestinal stress in mice, this experiment examined the expression levels of HSPs’ mRNA in the ileum. The results indicated a significant decrease in the mRNA expression level of HSP60 in the D group compared to other groups. The mRNA level of HSP90 was notably lower in the D group than that of the C group and G group, while the mRNA level of HSP70 did not significantly differ from that of the other groups. These findings align with those of previous studies [19]. Music can maintain intestinal immunity and alleviate stress response in mice without necessitating increased production of HSPs.

The intestine is the largest immune organ in the body, housing numerous immune cells and molecules diffused within the intestinal mucosal epithelium and lamina propria, collectively forming an intestinal immune barrier. B-lymphocytes are the main cells of intestinal immunity. When stimulated by antigens, B-lymphocytes proliferate and differentiate into plasma cells, which secrete abundant immunoglobulins to combat pathogens [29]. Among these immunoglobulins, IgA is the most abundantly produced class. Numerous studies have demonstrated that IgA, together with nonspecific immune proteins, constitutes the frontline defense of intestinal immunity by preventing microbial adhesion to intestinal epithelial cells, thus dampening intestinal inflammation. Additionally, IgA facilitates the establishment of gut-microbiota homeostasis. In instances where pathogenic bacteria breach the epithelial barrier, IgA, in synergy with IgG, works to eliminate them, thereby preserving intestinal health [30]. In this study, the mRNA levels of IgA in music groups were significantly higher than that in the C group, and the mRNA levels of IgG in the D group were significantly higher than those in other groups. Furthermore, the protein expression levels of IgG in the D group were significantly higher than those in the other groups. Mozart K.448 in D tone may strengthen intestinal immunity by promoting immunoglobulin production and supporting gut health. What is more, considering the results obtained regarding intestinal barrier function, intestinal permeability indices and HSPs, Mozart K.448 in D tone emerges as more conducive to the intestinal health of mice compared to A-tone and G-tone music.

Approximately 10^14^ microorganisms inhabit the human and animal gut, with diverse intestinal microbiota stimulating the mucosal epithelium to release immunoglobulins and mucins, crucial for the functioning of intestinal innate and adaptive immunity [31]. *Firmicutes* and *Bacteroidota*, as the two dominant phyla within the gut microbiota, exert profound influences on human health through their dynamic balance. Research indicates that *Firmicutes* play a crucial role in energy absorption and storage, whereas *Bacteroidota* are more adept at decomposing complex polysaccharides and participating in immune regulation [32]. An imbalance in the ratio of these two phyla is closely associated with a variety of metabolic disorders, such as obesity and type 2 diabetes, as well as inflammatory bowel disease (IBD). For instance, the gut microbiota of obese individuals often exhibit a higher relative abundance of *Firmicutes* and a lower proportion of *Bacteroidota*, a dysbiosis that may contribute to abnormal energy metabolism and chronic inflammation [33]. In healthy individuals, the abundance of *Proteobacteria* is relatively low; however, an increase in its proportion is often associated with gut dysbiosis and inflammatory conditions. Nevertheless, an appropriate level of *Proteobacteria* can contribute to the development and maturation of the intestinal immune system. By interacting with immune cells within the gut, *Proteobacteria* promote the secretion of immune factors and the differentiation of immune cells, thereby enhancing the host’s defense mechanisms against potential pathogens [34]. *Actinobacteria*, on the other hand, play a significant role in maintaining gut health and immune regulation. *Actinobacteria* are capable of fermenting dietary fibers to produce short-chain fatty acids (SCFAs), such as acetate and lactate, which help maintain an acidic intestinal environment, inhibit pathogen growth and support the health of epithelial cells. Additionally, *Actinobacteria* are involved in the synthesis of vitamins, such as vitamin B and K, which are crucial for the host’s nutritional metabolism [35]. Research indicates that significant alterations in the Firmicutes/Bacteroidota ratio may be directly associated with improvements in host metabolic phenotypes, while the stability of Proteobacteria suggests that the intervention did not compromise intestinal barrier function [36]. The experimental results of this study revealed that the ratio of *Firmicutes* to *Bacteroidota* was higher in the C group than music groups. This discrepancy may be associated with the musical environment to which the mice were exposed. Meanwhile, we also found that there were no significant differences in the abundances of *Proteobacteria* and *Actinobacteria* among the four groups. The experimental results of this study revealed that the ratio of Firmicutes to Bacteroidota was significantly higher in the control group compared to the music-exposed groups, suggesting that the musical environment may have modulated the gut microbiota composition in mice, potentially influencing host metabolic health. Further delving into the analysis at the genus level, the differences among the groups were more pronounced. For example, research by Mei et al. highlighted that *Lactobacillus* helps to establish intestinal microecological balance, reduces intestinal permeability damage caused by bacterial translocation and increases the level of IgA in the ileum and colon [37]. Consistent with Mei’s study, in the present study, we observed a higher abundance of *Lactobacillus* and an increased mRNA level of IgA in the ileum of the A group, accompanied by significantly decreased intestinal permeability. Hence, we posit that music may promote the colonization of beneficial intestinal bacteria, thereby influencing intestinal permeability and enhancing intestinal immunity. In addition, we also found that although the proportion of Lactobacillus in the A group was as high as 56.34%, significantly higher than that in the other groups, the abundances of *Paenalcaligenes*, *Oceanisphaera, Sporosarcina* and *Atopostipes* in the A group were all at an extremely low level and significantly lower than those in the other groups. These changes at the genus level reflect that specific musical stimulation (such as Mozart’s K448 in A tone) might have a selective effect on the growth and reproduction of certain microorganisms in the intestine, enabling them to proliferate abundantly or be inhibited. However, our study found no significant effect of music on the α-diversity and β-diversity of intestinal microbiota in mice, which aligns with the results of Chi’s study demonstrating that sound stimulation may not have a notable impact on the diversity and structure of the intestinal microbiota in mice [38]. Consequently, we suggest that music, as an auditory stimulus, can modulate the abundance of beneficial intestinal microbiota to prevent invasion caused by pathogenic bacteria and maintain the immune homeostasis of the intestinal mucosa. Overall, although different tones of Mozart’s K448 have a certain potential influence trend on the structure and diversity of the ileum microbiota in mice, it has not yet reached a significant degree of change. Future studies could consider prolonging the music intervention time, increasing the diversity of music types, or conducting comprehensive analyses in combination with other environmental factors to explore the complex relationship between music and the intestinal microbiota more comprehensively and deeply, providing a stronger theoretical basis for using music to regulate intestinal health.

## 4. Material and Methods

### 4.1. Animals and Experimental Groups

In this experiment, 100 one-day-old healthy male KM mice were housed at a temperature of 22 °C–25 °C under a 12 h light/dark cycle (with lights on at 8:00 a.m.), with a relative humidity of 50–60%. Mice had free access to commercial diets (crude protein ≥ 20%, crude fat ≥ 4%, crude fiber ≤ 5%, crude ash ≤ 8%, moisture ≤ 10%, calcium 1–1.8%, phosphorus 0.6–1.2%).

All KM mice were housed in groups of 5 per cage under controlled environmental conditions, including a 12 h light/dark cycle (lights on at 08:00, lights off at 20:00), with temperature maintained at 22 ± 1 °C and humidity at 50 ± 10%. The mice were randomly divided into four groups: control group (C group), D group, A group and G group. The C group did not receive music stimulation and served as a blank control. The D, A and G groups were exposed to music stimulation in the tones of D, A and G of Mozart K.448, respectively. All transposed versions were generated using Transcribe (9.21, Seventh String Software, Southampton, United Kingdom), and spectral validation was performed on transposed audio files to confirm the preservation of rhythmic patterns, dynamic ranges and timbral qualities without harmonic distortion.

The music stimulation was administered continuously from day 1 to day 63. The music groups were provided with left- and right-channel sound equipment, and music was played daily from 20:00 to 22:00 during the dark cycle to align with the natural activity period of the mice. The volume was maintained at 65 dB, and the position of the sound equipment and the intensity of the music remained constant throughout the stimulation period. All procedures employed in this experiment complied with the European Union Directive 2010/63/EU on the protection of animals used for scientific purposes and were approved by the Committee for the Protection and Use of Animals of the Northeast Agricultural University (No. SRM-06).

At 63 days of age, 15 mice were randomly selected from each group and immediately dissected upon sacrifice to collect ileal and its contents. The ileum was thoroughly rinsed repeatedly with ice-cold saline, and a portion of the ileal tissue and its contents were snap-frozen in liquid nitrogen and stored in a refrigerator at −80 °C for quantitative real-time polymerase chain reaction (qRT-PCR), Western blot, ELISA and 16S rDNA sequencing assays. The rest of the ileal tissue was fixed in 4% paraformaldehyde for hematoxylin and eosin (HE) staining and immunofluorescence analysis. Whole blood samples were centrifuged at 3000 rpm for 20 min at 4 °C to separate serum for determination of blood indices.

### 4.2. Hematoxylin and Eosin Staining

Ileal tissue was fixed in a 4% paraformaldehyde solution for 24 h. Subsequently, the tissue was gradually permeabilized with graded xylene and ethanol solutions to dehydrate. The tissue blocks were embedded in paraffin. Slices were obtained from each paraffin block using a microtome (Leica Biosystems, Nussloch, Germany), and then stained with hematoxylin and eosin. Images were taken using a 7800 Eclipse Ci-L orthogonal white light photomicrograph microscope (Nikon, Tokyo, Japan).

### 4.3. Immunofluorescence

After being deparaffinized, washed and dried, paraffin slices were dyed with fluorescent quenching reagent for 5 min and then washed with water for 10 min. Following this, primary antibodies including Claudin-1 (1:3000), Mucin2 (1:3000), Occludin (1:200) and ZO-1 (1:200, Servicebio, Wuhan, China) were added, and the slices were incubated overnight at 4 °C. The slices were treated with fluorescent secondary antibodies in the dark for 1 h. Subsequently, the slices were washed and decolorized in phosphate-buffered saline (PBS, pH = 7.4) on a slide shaker three times, each for 5 min. The slices were lightly dried and then treated with DAPI solution, followed by incubating at room temperature for 10 min and washing with PBS three times. After shaking the slices dry, a quench-resistant seal sheet with a fluorescent agent was applied. Once the slices were slightly dried, images were observed and captured under a fluorescence microscope. Image-Pro Plus 6.0 analysis software was utilized to calculate the positive area density (IOD/Area).

### 4.4. qRT-PCR

Total RNA was extracted from ileum using TRIZOL reagent (Invitrogen, Carlsbad, CA, USA). RNA quality and concentration were verified using a micro-spectrophotometer (Allsheng Instruments, Hangzhou, China). Once the tested value was qualified, the TAKARA RT kit was utilized to reverse transcription on the sample, resulting in a 1 μL sample containing 10 ng cDNA. The primers used in the experiment (Sangon Biotech, ShangHai, China) were diluted according to the primer instructions. The sequence of primers used for qRT-PCR are described in Table 1. The qRT-PCR reaction employed a 10 μL amplification system and was conducted on the LightCycler^®^ 480 System (Roche Diagnostics, Mannheim, Germany). The amplification system of qRT-PCR used is shown in Table 2. The relative mRNA levels were analyzed by the 2^−∆∆Ct^ method.

### 4.5. Protein Extraction and Western Blot

Following the above, 100 mg ileum was added to 1 mL cell lysis solution (Biosharp, Hefei, China) containing proteases and phosphatases (Beyotime, Shanghai, China), and then fully ground. Protein concentration was detected using BCA detection kits (Beyotime, Shanghai, China). Total protein was separated from tissues by 12% SDS-PAGE gel electrophoresis (Solarbio, Beijing, China) and then transferred to a polyvinylidene fluoride (PVDF) membrane (Cytiva, Marlborough, MA, USA). The PVDF membrane was blocked at 37 °C for 2 h and washed with phosphate-buffered solution (PBST) three times for 5 min per time. Subsequently, the primary antibody was incubated at 4 °C for 12 h, and washed in PBST three times for 15 min per time. Then, the second antibody was incubated at room temperature for 50 min and washed in PBST 3 times for 15 min. Enhanced chemiluminescence (ECL) reagent (Beyotime, Shanghai, China) was used for visualization by XX9 G:BOX Chemi imager (Syngene, Cambridge, UK). β-actin served as an internal reference standard for normalization. Information about the protein antibodies is provided in Table 3. Relative band intensities were quantified using ImageJ software (1.54f, National Institutes of Health, Bethesda, MD, USA).

### 4.6. Enzyme-Linked Immunosorbent Assay (ELISA) Detection

The levels of intestinal permeability index (Endotoxin, DAO, D-lactate) in serum and the levels of immune globulin (IgA, IgG) in ileum tissue were measured with corresponding ELISA kits according to the manufacturer’s instructions (Xinle, Shanghai, China).

### 4.7. 16S rDNA Sequencing and Bioinformatics Analysis

Using the specific kit for total DNA extraction from intestinal contents (E.Z.N.A.^®^ Stool DNA Kit, Omega Bio-tek, Norcross, GA, USA), the total DNA of the microbiome in the ileum contents of mice was extracted. The quality of DNA in each sample was detected by agarose gel electrophoresis. Meanwhile, the concentration of the total DNA in the samples was detected using an ultraviolet spectrophotometer (Thermo Fisher Scientific, Waltham, MA, USA). Then, the total DNA samples were diluted with 50 microliters of ultrapure water and placed in an −80 °C ultra-low temperature freezer for standby. Based on the V3-V4 hypervariable region sequence of microbial 16S rDNA, specific universal primers with barcode sequences (341F: 5′-CCTACGGGNGGCWGCAG-3′ and 805R: 5′-GACTACHVGGGTATCTAATCC-3′) were designed and synthesized for PCR amplification. The PCR reaction system is detailed in Table 4. The PCR amplification conditions were as follows: pre-denaturation at 98 °C for 30 s, denaturation at 98 °C for 10 s, annealing at 54 °C for 30 s, extension at 72 °C for 45 s and 32 cycles. The final extension was at 72 °C for 10 min.

The PCR product was purifed using AMPure XP Beads (Beckman Coulter Genomics, Danvers, MA, USA) and quantifed using Qubit (Invitrogen, Carlsbad, CA, USA). Qualifed PCR products were evaluated using an Agilent 2100 Bioanalyzer (Agilent, Santa Clara, CA, USA) and Illumina library quantitative kits (Kapa Biosciences, Woburn, MA, USA), which were further pooled together and sequenced on an Illumina NovaSeq 6000 (PE250), provided by LC-Bio Technology Co., Ltd., Hangzhou, China.

Sequencing primers were removed from the demultiplexed raw sequences using Cutadapt (version 1.9). Subsequently, paired-end reads were merged using FLASH (version 1.2.8). The low-quality reads (with quality scores < 20), short reads (<100 bp) and reads containing more than 5% “N” characters were trimmed by applying the sliding-window algorithm in fqtrim (version 0.94). Quality filtering was carried out based on fqtrim to obtain high-quality clean tags. Chimeric sequences were filtered using Vsearch software (version 2.3.4). DADA2 was employed for denoising and generating amplicon sequence variants (ASVs). The sequence alignment for species annotation was performed using the QIIME2 plugin feature-classifier. The alignment databases used were SILVA (version 138.1) and NT-16S, with SILVA given higher priority. The results from both databases were combined to generate the final species annotation, which was then used for downstream analyses. Alpha and beta diversities were calculated using QIIME2. Relative abundance was used in bacteria taxonomy. The Wilcox test was used to identify genera with significant differences, and significance was declared when *p* < 0.05. The Linear Discriminant Analysis Effect Size (LEfSe, with LDA ≥ 3.0 and *p* < 0.05) was performed using nsegata—lefse. Other diagrams were generated using the R package (version 3.4.4).

### 4.8. Statistical Analyses

The IBM SPSS Statistics (26.0, SPSS Inc., Chicago, IL, USA) was employed to analyze the data. One-way analysis of variance was utilized to examine the changes in Alpha diversity indices and the abundances of dominant microbiota at the phylum, order, family and genus levels among groups. Moreover, Duncan’s multiple comparison test was applied to compare the significance of differences in each index. During the LEfSe analysis of the differences in gut microbiota abundances between two groups, the Kruskal–Wallis rank sum test was used to compare the differences of microbiota among different taxonomic levels between groups, and the LDA score was adopted to evaluate the degree of influence. When *p* < 0.05 and LDA score > 3.0, it was considered that there was a significant difference in the abundances of microbiota between groups. For the relevant data of ELISA, mRNA and Western Blot, prior to statistical analysis, all the obtained data were first subjected to the Kolmogorov–Smirnov test for normality distribution. After confirming that all the data in this experiment conformed to the normal distribution, multivariate analysis of variance under the general linear model was used to analyze the data between treatment groups, and Duncan’s multiple test was utilized to compare the differences between treatment groups. The results were expressed as mean ± standard deviation (mean ± SD). *p* < 0.05 was regarded as statistically significant.

## 5. Conclusions

Musical stimulation, especially Mozart’s K448 in D tone, can enhance the expression of intestinal barrier genes to maintain intestinal structural integrity, thereby reducing intestinal permeability and sustaining intestinal immune function. Furthermore, musical stimulation did not significantly alter the diversity of intestinal microbiota. However, it led to an increase in the abundance of beneficial bacteria and a decrease in harmful bacteria at both the phylum and genus levels. These results reveal a potential mechanism by which Mozart’s K448 maintains intestinal health and promotes intestinal microbiota homeostasis in mice.

## Figures and Tables

**Table 1:** Sequence of primers used for qRT-PCR.

Gene	Serial Number	Primer Sequence (5′-3′)
Occludin	NM_001360536.1	F: TTGGCTACGGAGGTGGCTATGG R: CCTTTGGCTGCTCTTGGGTCTG
Claudin-1	NM_016674.4	F: GCTGGGTTTCATCCTGGCTTCTC R: CCTGAGCGGTCACGATGTTGTC
Claudin-12	NM_001193659.1	F: GTCCTGTCCTTCCTGTGTGGTATTG R: CAGCCGCAGTTTCCTCCAGTTAG
Mucin2	NM_023566.4	F: CGAGCACATCACCTACCACATCATC F: CGAGCACATCACCTACCACATCATC
ZO-1	NM_001163574.2	F: CATGTCTCTAACGGATGCTCGGAAG R: GTTTAGGGCTGGGATGTTGATGAGG
ZO-2	NM_001198985.2	F: AACCCGAAACTGATGCTGTGGATAG R: CGCCCTTGGAATGTATGTGGAGAG
IgA	NM_007655.4	F: GCGAGCAGACTCAGCCAAGAAG R: AGCCACACCACCCACCACTC
IgG	NM_005838.1	F: AGAAGTTCAAGAGCAAGGCCACAC R: ACCGCAGAGTCCTCAGATGTCAG
HSP60	NM_001356512.1	F: CACCACCACTGCCACTGTTCTG R: TACAGCATCCACAGCCAACATCAC
HSP70	NM_010478.2	F: GGTGCTGACGAAGATGAAGGAGATC R: CTGCCGCTGAGAGTCGTTGAAG
HSP90	NM_008302.3	F: GGCTGAGGACAAGGAGAACTACAAG R: GGCTGAGGACAAGGAGAACTACAAG
β-actin	NM_007393.5	F: CTGAGAGGGAAATCGTGCGTGAC R: ACCGCTCGTTGCCAATAGTGATG

**Table 2:** Amplification system of qRT-PCR.

Reagent	Volume
FastStart Universal SYBR Green Master (ROX)	5.0 μL
Forward primer	0.3 μL
Reverse primer	0.3 μL
DEPC water	3.4 μL
cDNA	1.0 μL

**Table 3:** Protein antibody information.

Antibody Name	Dilution Ratio	Manufacturer of Products
β-actin	1:5000	ABclonal
Claudin 1	1:1500	Wanleibio
Claudin 5	1:1000	Wanleibio
Occludin	1:1000	Wanleibio
G/R IgG-HRP	1:40,000	Bioss Antibodies

**Table 4:** Amplification system of PCR.

Reagents	Volume
Phusion Hot start flex 2×Master Mix	12.5 μL
Primers upstream of the target gene	2.5 μL
Primers downstream of the target gene	2.5 μL
cDNA	50 ng
Make up ddH_2_O to	25 μL

## Data Availability

The data supporting the findings of this study are available within the article. No new datasets were generated or analyzed during this study.
